# Analysis of the Steelmaking Process via Data Mining and Pearson Correlation

**DOI:** 10.3390/ma17112786

**Published:** 2024-06-06

**Authors:** Susana Carrasco-López, Martín Herrera-Trejo, Manuel Castro-Román, Fabián Castro-Uresti, Edgar Iván Castro-Cedeño

**Affiliations:** 1 Centro de Investigación y de Estudios Avanzados, CINVESTAV Saltillo, Av. Industria Metalúrgica No. 1062, Parque Industrial Saltillo-Ramos Arizpe, Ramos Arizpe 25900, Coahuila, Mexico; susana.carrasco@cinvestav.edu.mx (S.C.-L.); manuel.castro@cinvestav.edu.mx (M.C.-R.); edgar.castro@cinvestav.edu.mx (E.I.C.-C.); 2 Ternium México, San Nicolás de los Garza 66450, Nuevo León, Mexico; fcastro@ternium.com.mx

**Keywords:** data mining, decision tree classifier, Pearson correlation, refining steelmaking

## Abstract

The continuous improvement of the steelmaking process is a critical issue for steelmakers. In the production of Ca-treated Al-killed steel, the Ca and S contents are controlled for successful inclusion modification treatment. In this study, a machine learning technique was used to build a decision tree classifier and thus identify the process variables that most influence the desired Ca and S contents at the end of ladle furnace refining. The attribute of the root node of the decision tree was correlated with process variables via the Pearson formalism. Thus, the attribute of the root node corresponded to the sulfur distribution coefficient at the end of the refining process, and its value allowed for the discrimination of satisfactory heats from unsatisfactory heats. The variables with higher correlation with the sulfur distribution coefficient were the content of sulfur in both steel and slag at the end of the refining process, as well as the Si content at that stage of the process. As secondary variables, the Si content and the basicity of the slag at the end of the refining process were correlated with the S content in the steel and slag, respectively, at that stage. The analysis showed that the conditions of steel and slag at the beginning of the refining process and the efficient S removal during the refining process are crucial for reaching desired Ca and S contents.

## 1. Introduction

The reproducibility of the steelmaking process is an essential issue for steelmakers because of the interest in ensuring the standardization of final requirements in a systematic way. Thus, it is desirable to identify areas of opportunity by privileging formal procedures over heuristic procedures that, when adequately addressed, can contribute to improving the quality of products. Generally, numerous process parameters are recorded throughout the steelmaking process. This information is stored in a database that can be statistically exploited to prioritize the influential variables in the control process that lead to the desired quality requirements.

In Al-killed steels, the presence of solid Al_2_O_3_ inclusions as the main product of the deoxidation reaction is generally undesirable. The harmful effect of Al_2_O_3_ inclusions is avoided by inclusion modification by Ca; Ca is dissolved through the injection of cored Ca wire and reacts with solid Al_2_O_3_ inclusions to form liquid Ca aluminates with a low melting point. This promotes their flotation to the top slag and decreases the adverse effects of the remaining inclusions [1]. Successful Ca treatment, i.e., the formation of fully liquid Ca aluminate, depends on the Al, O, S, and Ca contents in the steel. Gaye et al. [2] illustrated the effect of Al and Ca on the Ca aluminate type to be formed and the associated limit of the S content to avoid the undesirable precipitation of CaS. The control of the sulfur content is related to the Al deoxidation process through the desulfurization reaction [3]:

(1) $${\underline{S}}_{metal}+{\left(O^{2-}\right)}_{slag}={\underline{O}}_{metal}+{\left(S^{2-}\right)}_{slag}$$

where the underlined characters and parentheses denote the species dissolved in the steel and the species dissolved in the slag, respectively. Hence, a low sulfur content is promoted by a high basicity of slag (wt pct CaO/wt pct SiO_2_) and a low content of O. Although the thermodynamic fundamentals for S removal are known, its efficiency depends on the control of the variables involved in the heterogeneous kinetics of the desulfurization reaction.

Recently, Miao et al. [4] conducted a study to analyze the Ca inclusion modification in plant-scale heats. In their analysis, the authors considered steel chemistry-based parameters proposed in the literature as guidelines to characterize the inclusion modification treatment, and as a result, they proposed a new parameter based on the Al, Ca, and S contents in the steel. A recent study showed that the use of statistical tools represents an alternative to the analysis of inclusion modification by Ca treatment. De Sousa et al. [5] analyzed a plant database and developed a statistically significant model via multiple linear regression to identify the main variables to be controlled for successful Ca treatment. The model indicated that the oxidation of the steel and the S and Ti contents in the steel were the main influential variables and enabled the design of process strategies leading to the better control of the inclusion modification.

On the other hand, machine learning techniques have recently been used to analyze and improve the steelmaking control process [6,7,8]. The selected models differ in their degree of sophistication and scope. Among machine learning models, the rule-based classifier model is quite widespread because it generates easily interpretable rules. Recently, Oliver and Aldrich [9] used a rule-based classifier model to construct a decision tree to support a rule-based decision system for grinding circuits. Their approach was able to identify the most influential process variables in the control of the process. Although this approach is well known and has several applications, it has scarcely been used for the analysis of steelmaking processes.

In the present work, a decision tree classifier was constructed to identify the most influential process variables for obtaining suitable final Ca and S contents for the successful production of Ca-treated Al-killed steel. The root node attribute corresponded to the main influential variable, and the effects of other variables were associated with the internal and leaf nodes. The characteristics of those variables allowed us to discriminate satisfactory heats from unsatisfactory heats. Additionally, the correlation of the root node attribute with the total number of variables was evaluated. The variables with higher correlation were analyzed to identify opportunity areas for the control process.

## 2. Description of the Process

**Figure 1:**
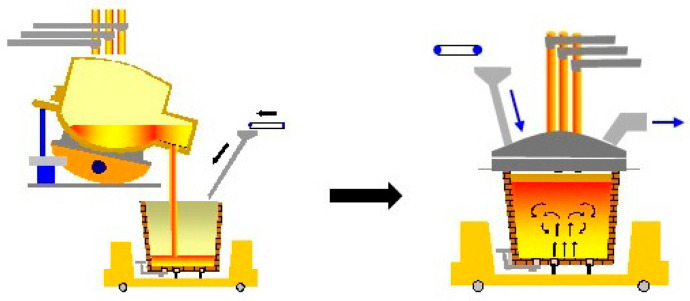
Processing route of Ca-treated Al-killed steel.

The steelmaking process under analysis involved the production of Ca-treated Al-killed steel via an electric arc furnace (EAF)–ladle furnace (LF) refining route, as shown in Figure 1. The primary steel in the EAF was produced by using direct reduced iron, scrap, and the remaining hot heel in the furnace. After the charge melted, the chemical composition and temperature were adjusted, and the steel was tapped into a 150-ton ladle. During this operation, Al, slag-forming agents, and ferroalloys were added to the stream of liquid steel to initiate the desired reactions, such as deoxidation and desulfurization. After tapping, the ladle was taken to the LF station, and the refining process started with the homogenization of both the chemical composition and temperature of the liquid steel, and the simultaneous conditioning of the slag. The properties of the slag, such as basicity (B = %CaO/%SiO_2_) and oxidation level (%FeO + %MnO), were adjusted to promote sulfur removal through the steel-top slag reaction, and the chemical composition of the metal was simultaneously adjusted. Weight percent (%) is used to denote chemical composition throughout this paper. Once the desired contents of the alloy elements, as well as Al and S, were reached, the liquid steel was subjected to Ca inclusion modification treatment through the injection of a CaFeAl wire. The LF refining ended with a period of gentle stirring to promote the transport of inclusions to the steel–slag interphase, and then the ladle was transferred to the continuous casting caster.

## 3. Methodology

A statistical analysis based on a database of steelmaking process variables was performed to identify those that most influenced the desired S and Ca contents at the end of LF refining to <0.005% and >0.0024%, respectively, in the production process of Al-killed steel. The selection of those content values was based on metallurgical analysis and plant experience, as they have been shown to avoid subsequent complications during the process and ensure successful Ca treatment. Table 1 shows the typical chemical composition of the analyzed steel.

First, a reliable database was developed from the plant records of process variables. The records included data from the EAF tapping process to the end of the LF refining process. The initial database was cleaned by removing heats with incomplete records and/or measurement errors, and the resulting database included 530 heats.

A descriptive statistical analysis using Python 3.8 software [10] was performed to remove data without physical realistic meaning, and then the database was supplemented with calculations of metallurgical variables derived from recorded variables. Thus, variables such as B, the oxidation level of slag, and the experimental sulfur distribution coefficient (Ls_(exp)_) were calculated at the initial and final stages of the refining process; the subscripts i and f are used hereafter to denote the initial stage and final stage, respectively, of the refining process. Notably, Ls is given by the quotient between the sulfur content in the slag (%S) and the sulfur content in the liquid steel %S. Additionally, data were used to calculate the sulfur capacity (Cs) and the theoretical sulfur distribution coefficient (Ls_(theo)_) by using the thermodynamic KTH model developed by Sheetaraman et al. [11].

Once the database was supplemented, it was used to calculate a decision tree classifier to identify the attributes of the root node, internal nodes, and leaf nodes; Orange 3.31.1 software [12] was employed. Ninety-six parameters were selected as the main variables, and the target values of %S and %Ca were specified. The number of splits by the leaf nodes was set to 2, i.e., the tree became if-then-else conditional, the maximum depth was set to 90% to avoid overestimations, and the improvement threshold value was greater than 80%. To correlate the attribute of the root node with the process variables, the Pearson correlation procedure [13] was performed; thus, the variables with greater correlation were identified.

## 4. Results and Discussion

**Figure 2:**
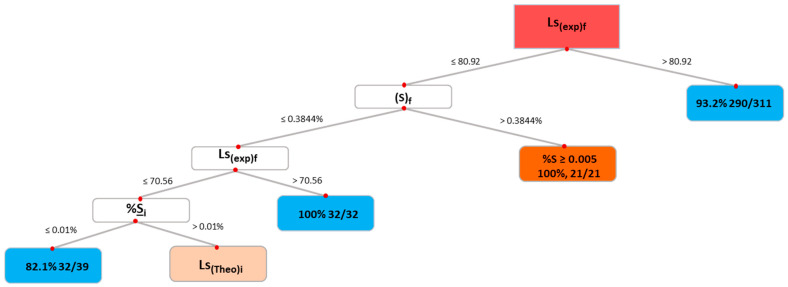
Decision tree classifier.

The obtained decision tree classifier is shown in Figure 2. The attribute of the root node, shown in red, was Ls_(exp)f_, and for the first three levels the nodes shown in blue correspond to (1) Ls_(exp)f_ > 80.92, (2) 70.56 < Ls_(exp)f_ < 80.92, and (3) Ls_(exp)f_ ≤ 70.56. At the first level, when Ls_(exp)f_ > 80.92, 290 heats of 311 (93.2%) were satisfactory. For the second level, the final S content in the slag, (S_f_) ≤ 0.3844% and Ls_(exp)f_ > 70.56 were needed, and the number of heats was 32 of 32. If the condition of (S_f_) ≤ 0.3844% was not met (21 heats), the final sulfur content in the metal, %S_f_, was higher than 0.005%, and the heats were unsatisfactory. For the third level, the initial content of S in the metal of %S_i_ ≤ 0.010 was needed, and 32 heats of 39 met that condition; in the case of %S_i_ > 0.010, satisfactory heats required specific values of three consecutive parameters, which are not included in the figure because of the decreased number of heats involved: Ls_(theo)i_, Ls_(exp)f_, and (S)_f_. The first three levels of the decision tree only were analyzed because they represented 70% of the heats.

**Figure 3:**
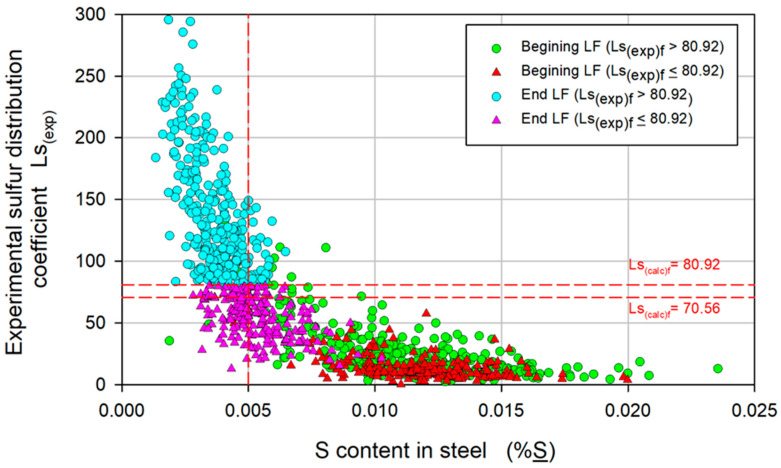
Variation in the experimental sulfur distribution coefficient, Ls_(exp)_, with the S content in the steel, %S.

Figure 3 shows the variation in Ls_(exp)_ with %S to illustrate the three upper levels of the decision tree. %S was selected for the analysis rather than Ca because it is a parameter that is controlled in plants from the early stages of the steelmaking process. Dashed horizontal lines are included in the figure to indicate the values of Ls_(exp)_ of 80.92 and 70.56, and a vertical line indicates the maximum permissible limit of %S_f_, 0.005. In most of the following figures, data corresponding to the beginning of the LF refining were included for analysis purposes and were labeled based on Ls_(exp)f_ values, i.e., those that reached values greater than 80.92 at the end of LF refining and those that did not.

Considering the limit of %S_f_ < 0.005, the largest number of heats is shown in the upper left area of the figure, corresponding to the first level of the decision tree: Ls_(exp)f_ > 80.92. The number of heat events decreased for the second level, at 70.56 < Ls_(exp)f_ < 80.92, and for the third level, at Ls_(exp)f_ ≤ 70.56. Not all the heat events found in the corresponding zone were satisfactory; only those with S_f_ ≤ 0.01 were satisfactory. Furthermore, the heats with Ls_(exp)f_ > 80.92 presented higher values of Ls_(exp)i_ than those with Ls_(exp)f_ < 80.92, which indicates that the initial conditions in the refining process, i.e., %S_i_ and Ls_(exp)i_, and consequently (%S)_i_, are essential for obtaining the desired values of %S_f_. The initial conditions result from the removal of sulfur during the tapping process; sulfur removal is promoted by the low activity of O and the high basicity of slag in accordance with reaction (1). The O content and B are controlled by primary deoxidation with Al and the addition of slag-forming agents, respectively. However, the control of both parameters is hindered by the numerous variables involved and the stochastic nature of the tapping process, which causes variability in the initial conditions for the removal of S in LF refining.

**Figure 4:**
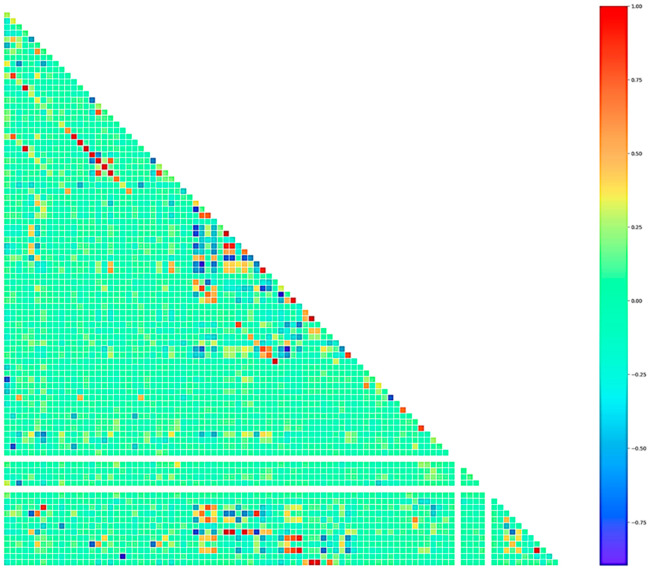
“Heatmap” for the estimated Pearson correlation.

To analyze the correlation of the first level of the decision tree, Ls_(exp)f_ > 80.92, with the processing variables (69), Pearson’s formalism was utilized. Figure 4 shows the results of the estimated correlation in heatmap format, where red indicates a high positive correlation and dark blue indicates a high negative correlation; the labels of the variables on the axes are not included in the figure because of the reduced relationship space/number of variables. Table 2 shows the main variables with the highest correlation, and the secondary variables are correlated with the main variables. The main variables were S_f_, Si_f_, and (S)_f_, and Si_f_ and B_f_ were correlated as secondary variables to S_f_ and (S)_f_, respectively.

**Figure 5:**
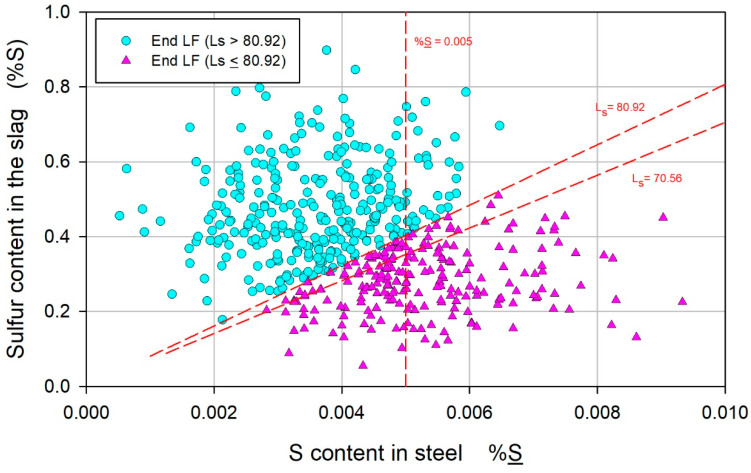
Correlation of the sulfur content in the slag (%S) with the sulfur content in the steel (S).

It was reasonable that the contents of S_f_ and (S)_f_ were correlated with Ls_(exp)f_ since those variables were used to calculate this parameter. Figure 5 shows this correlation, where (%S) is plotted as a function of %S, the Ls_(exp)f_ lines are 80.92 and 70.56, and the line corresponding to the satisfactory limit of %S is included. For a given acceptable value of %S < 0.005, the requirement for a satisfactory value of Ls_(exp)f_ is a sufficiently high value of (%S). A high value of (%S)_f_ suggests, on the one hand, that during LF refining, the slag is sufficiently chemically conditioned and, on the other hand, that the kinetic conditions for sulfur removal are favorable, in addition to low and high values of %S_i_ and Ls_(exp)I_, respectively, as shown in Figure 3.

**Figure 6:**
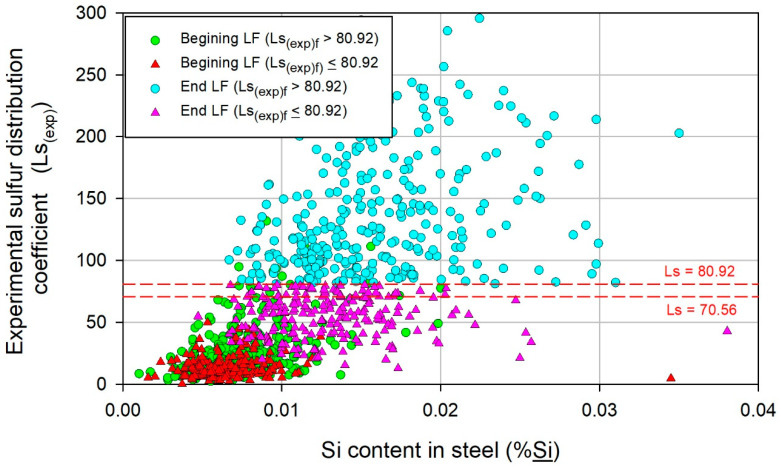
Variation in Ls_(exp)_ with the Si content in the steel (%Si).

The correlation of Si_f_ with Ls_(exp)f_ is shown in Figure 6; the general trend is the increase in Ls_(exp)_ with %Si, and the Ls_(exp)i_ values where the Si contents are low tend to be higher for heats with Ls_(exp)f_ > 80.92, thus emphasizing that the better the initial state is, the better the final state. Furthermore, for a given %Si at the end of LF refining, the value of Ls_(exp)f_ varies over a wide range, which could be associated with both the variability of Ls_(exp)i_ with %Si_i_ and operative variations throughout the LF refining process.

**Figure 7:**
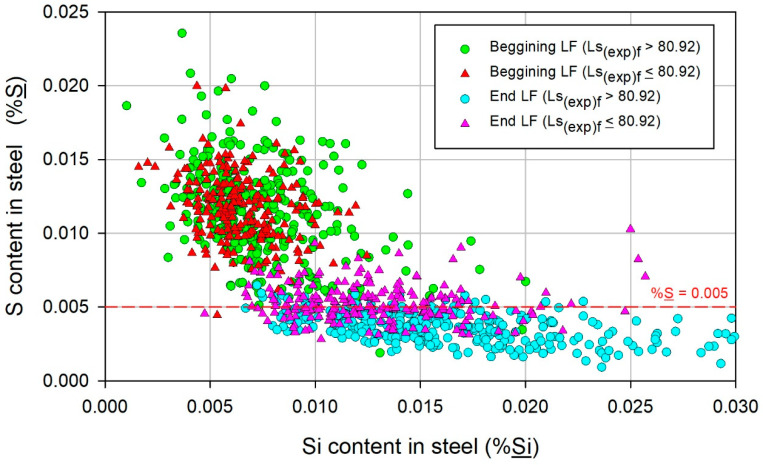
Variation in the S content in steel, %S, with the Si content in the steel, %Si.

The correlation of Si_f_ as a secondary variable with S_f_ is shown in Figure 7. At the beginning of LF refining, no distinct trend could be deduced because of the scattering of the data, and the %S was greater than 0.005. At the end of LF refining, the %S decreased with increasing %Si for heats with Ls_(exp)f_ > 80.92, whereas for heats with Ls_(exp)f_ < 80.92, the effect of Si was not distinct. The effect of Si on promoting the removal of S has been reported in plant-scale heats [14,15], as well as in studies that analyze it through mathematical simulations that involve transient thermodynamic and kinetic conditions [14,16]. It has been shown that Si acts by decreasing the activity of O in the adjacent volume at the liquid steel–slag interface, which is promoted by the high activity of Si and low activity of SiO_2_. Thus, Al is prevented from reducing the oxides present in the slag and consequently remains available to promote the desulfurization reaction. Thus, the efficient role of Si implies a reduction in the presence of reducible oxides in the slag.

**Figure 8:**
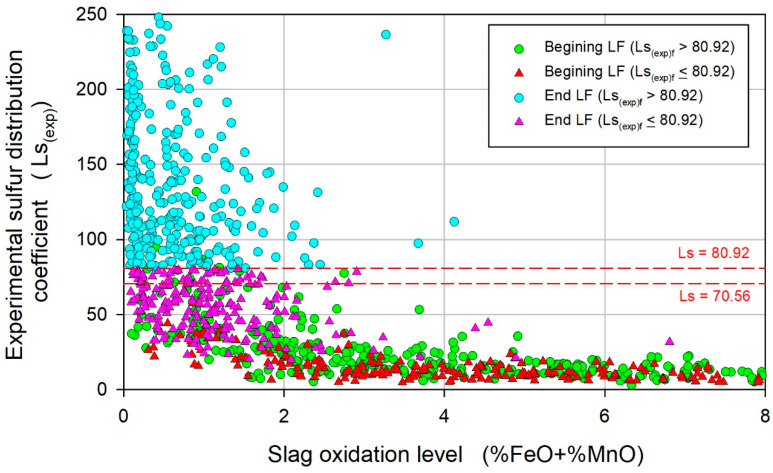
Variation in Ls_(exp)_ with respect to slag oxidation level (%FeO + %MnO).

The effect of the reducible oxides in the slag (%FeO + %MnO) on Ls_(exp)_ is shown in Figure 8. In general, relatively little variation and insufficient values of Ls_(exp)_ were observed at the beginning of LF refining, despite the great variation in (%FeO + %MnO), which is associated with the difficulty in quickly forming an effective slag for sulfur removal, which is related to the complex and stochastic nature of the tapping process. At the end of LF refining, the (%FeO + %MnO) parameter tended toward low values, and the average values for heats exhibiting Ls_(exp)f_ > 80.92 and Ls_(exp)f_ ≤ 80.92 were 0.64 ± 0.06 and 1.5 ± 0.14, respectively. Regardless of the difference between the average values, for a given low (%FeO + %MnO) value, the Ls_(exp)f_ varies over a wide range, suggesting, based on the variability of the initial conditions, that for satisfactory heats, a lower oxidation level of the slags is promptly established during LF refining than for unsatisfactory heats. Thus, the initial condition of the slag and its evolution, as well as the heterogeneous kinetics of sulfur removal, must be considered to improve the control of the removal of S.

**Figure 9:**
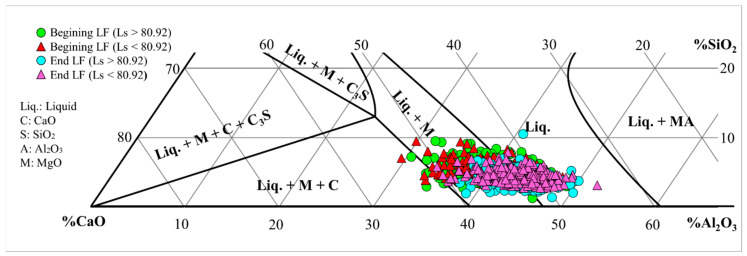
Chemical composition of slags in the Al_2_O_3_-CaO-SiO_2_-10%MgO diagram at 1873 K.

On the other hand, to analyze the correlation of B_f_ as a secondary variable with (S)_f_, the chemical compositions of the slags were adjusted by decreasing their supersaturation in CaO and thus avoiding overestimation. For fitting, the Al_2_O_3_-CaO-SiO_2_-10%MgO ternary diagram was calculated using Thermocalc 2018b software [17], and then the chemical compositions were plotted. Figure 9 shows that the chemical compositions of the slags were corrected to bring them closer to the saturation limit.

**Figure 10:**
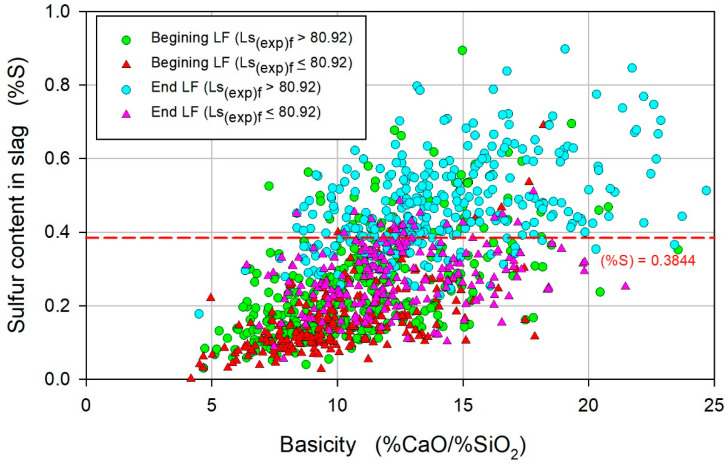
Variation in the sulfur content in the slag (%S) with the basicity of the slag.

Figure 10 shows the correlation of (%S) with B, where an increase in (%S) with B can be observed regardless of the processing stage. Furthermore, it was observed that the (%S)_i_ values were higher for the heats with satisfactory (%S)_f_ values: the average values of (%S)_i_ were 0.24 ± 0.002 and 0.1% ± 0.004 for heats with satisfactory values and unsatisfactory (%S)_f_ values, respectively. Thus, if (%S)_i_ > 0.24, a satisfactory heat will be expected, which indicates that the initial conditions in LF refining, i.e., (%S)_i_, are critical for reaching a satisfactory value of (%S)_f_. The differences in (%S)_i_ values are associated with variations in the amount of sulfur removal during the tapping operation and the time available for the sulfur removal. Obtaining adequate slag for sulfur removal during the tapping operation is complex and stochastic, and depends on numerous variables, such as the quantity and moment of the addition of slag agents, deoxidizing agents, and deoxidation products, as well as stirring intensity and the flow pattern of the metal–slag system. Regarding the time available for the sulfur removal, the time involved in the transport of the ladle is considered important for the desulfurization heterogeneous reaction, which is favored by the increased thermodynamic driving force existing in that stage, which is associated with the difference in the sulfur chemical potential between the steel and the slag.

**Figure 11:**
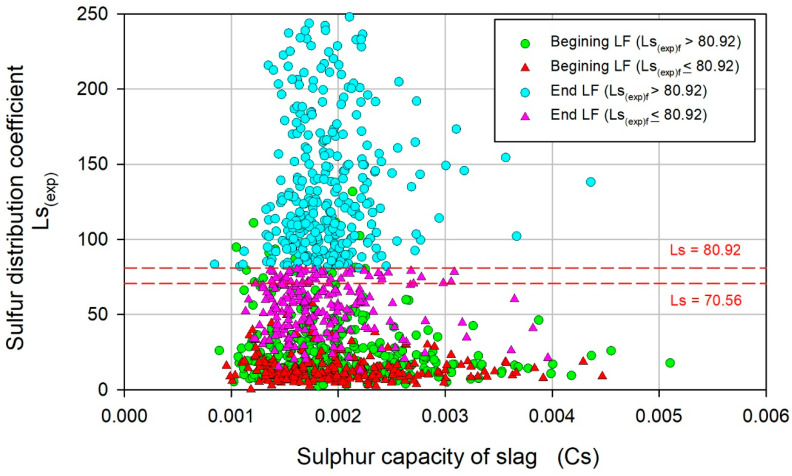
Variation in Ls_(exp)_ with respect to the sulfur capacity of slag Cs.

To further elucidate the role of slag in the removal of sulfur, Cs was considered. Cs is a property that represents the potential ability of slag to dissolve sulfur and depends only on the chemical composition and temperature. Figure 11 shows the variation in Ls_(exp)_ with Cs. For unsatisfactory heats, the average values of Cs at the beginning and end of LF were 0.0014 and 0.0016, respectively, while for satisfactory heats, those values were 0.0022 and 0.0019, indicating that in satisfactory heats, the slag was better controlled from the beginning of LF refining. Thus, if Cs ≥ 0.0022 at the beginning of LF refining, a satisfactory value of Ls_(exp)f_ is expected. Furthermore, for given values of Cs in the range of satisfactory heats, Ls_(exp)f_ varies over a wide range, which suggests that the evolution of S and (S) depends not only on the thermodynamic conditions imposed by Cs, but also on the kinetic conditions associated with the transfer of S to the metal–slag interface, where the desulfurization reaction occurs.

**Figure 12:**
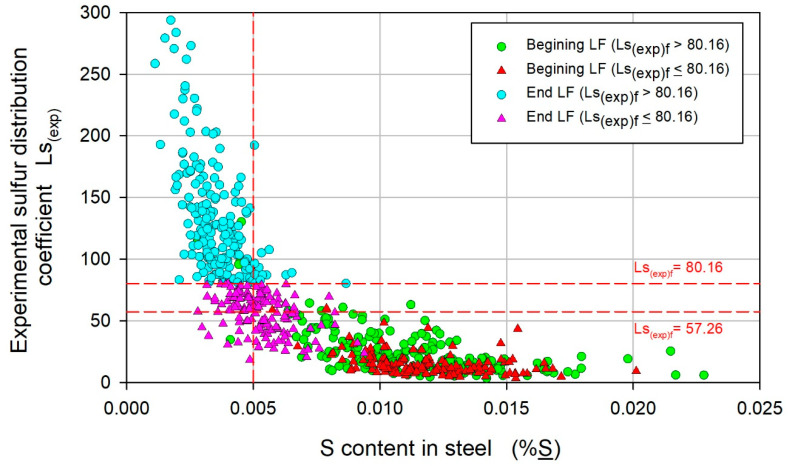
Variation in the experimental sulfur distribution coefficient, Ls_(exp)_, with the S content in the steel %S for the second steel.

The methodology used in the present work was replicated for another steel whose chemical composition was similar to that of the analyzed steel, and the results are presented in Figure 12, which is a graphic representation of the decision tree. From a comparison of this figure with that of the analyzed steel (Figure 3), a similar general behavior is deduced. The Ls_(exp)_ values for the first level of the decision tree were similar, while for those of the second level, the difference was more appreciable, at 70.56 and 57.26 for the first steel and second steel, respectively. This finding indicates that this methodology can be used as an additional tool for process analysis since it can identify the variables that most influence desirable Ca and S contents, and provide their critical values.

## 5. Conclusions

A machine learning technique was used to build a decision tree classifier and thus determine the process variable that most influences the desired Ca and S contents at the end of ladle furnace refining in the production of Ca-treated Al-killed steel. The attribute of the root node of the decision tree was correlated with the process variables via the Pearson formalism. Thus, the following conclusions are drawn:

- The methodology used can serve as a tool for process analysis. This approach allowed us to identify the main variables influencing the desired Ca and S contents at the end of ladle furnace refining and to statistically estimate their critical values.
- The methodology showed coherence with the existing metallurgical background, which constitutes an opportunity to deepen our understanding of the process and improve it accordingly.
- The analysis showed that the conditions of the steel and slag at the beginning of the refining process are decisive for reaching the desired contents of Ca and S in the steel at the end of the refining process.

## Figures and Tables

**Table 1:** Typical chemical composition of the analyzed steel.

%	Element												
	C	Si	Mn	S	Al	Ca	Mo	Ni	P	Cu	B	Ti	V
Min	0.03	-	0.1	-	0.02	0.001	-	-	-	-	0.0025	-	-
Max	0.06	0.044	0.2	0.015	0.05	-	0.02	0.1	0.018	0.12	0.007	0.006	0.008

**Table 2:** There were higher Pearson correlation coefficients for Ls_(exp)f_ with the process variables.

Main Variables	Correlation Coefficient	Secondary Variables	Correlation Coefficient
S_f_	−0.62	Si_f_	−0.54
Si_f_	0.51		
(S)_f_	0.39	B_f_	0.45

## Data Availability

Data are contained within the article.
